# Vortioxetine as a potential alternative for patients with escitalopram‐induced jitteriness/anxiety syndrome: A report of three cases

**DOI:** 10.1002/pcn5.158

**Published:** 2023-11-16

**Authors:** Kaoru Arai, Mari Nonaka, Shoko Shimada, Masayuki Nakamura

**Affiliations:** ^1^ Department of Psychiatry Kagoshima University Graduate School of Medical and Dental Sciences Kagoshima Japan

**Keywords:** depression, escitalopram, jitteriness/anxiety syndrome, selective serotonin reuptake inhibitor, vortioxetine

## Abstract

**Background:**

Jitteriness/anxiety syndrome is a recognized adverse effect observed during the initiation or change of dose in antidepressant treatment. Managing patients who develop this syndrome remains a challenge. While escitalopram is a widely used antidepressant known to cause these symptoms, this report explores vortioxetine as a therapeutic alternative.

**Case Presentation:**

Three distinct clinical scenarios were observed in patients who manifested jitteriness/anxiety syndrome while on escitalopram treatment for depression. Patient A was initiated on escitalopram and experienced an initial alleviation in depressive symptoms, but 3 months later displayed mood elevation, talkativeness, and increased activity, which disturbed his daily life. A transition to vortioxetine subsequently resolved the mood elevation. Patient B exhibited elevated mood, hyperactivity, irritability, and talkativeness just 6 days post‐initiation of treatment with escitalopram. After the discontinuation of escitalopram and unsuccessful trials with aripiprazole, lurasidone, and lamotrigine, her depressive mood intensified, culminating in suicidal ideation. Starting vortioxetine led to a consistent improvement of her symptoms, and she resumed work and was emotionally stable. Patient C was initially diagnosed with bipolar disorder and faced a relapse into depression despite undergoing various treatments. After 2 weeks on escitalopram, she exhibited irritability and self‐harm urges. Three months later, after being re‐diagnosed with depressive disorders with anxious distress, vortioxetine was administered, which significantly reduced her depressive symptoms and allowed her to continue her education.

**Conclusion:**

Vortioxetine presents as a promising therapeutic alternative that is worth considering for patients with escitalopram‐induced jitteriness/anxiety syndrome.

## Graphical abstract

Managing patients who develop antidepressant‐induced jitteriness/anxiety syndrome remains a challenge. Our observations suggest that vortioxetine might offer a safer and more efficacious alternative in such circumstances, especially when the discontinuation of antidepressant therapy is not a suitable option.

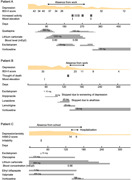

## BACKGROUND

The efficacy of antidepressants in the treatment of depression is widely accepted, especially for moderate to severe levels of major depressive disorder.^1^, ^2^ However, adverse events associated with the use of antidepressants constitute a major clinical problem. The emergence of symptoms such as anxiety, agitation, panic attacks, insomnia, irritability, hostility, aggressiveness, impulsivity, akathisia, hypomania, and mania following the administration of antidepressants has frequently been observed and was defined as “activation syndrome” in a 2004 United States Food and Drug Administration warning.^3^ Later, the term “jitteriness/anxiety syndrome” was proposed.^4^, ^5^ However, the lack of a clear definition or an established treatment for this syndrome^4^ undermines the treatment of depression.

Vortioxetine is a multimodal antidepressant with a unique receptor profile as a serotonin (5‐hydroxytryptamine [5‐HT]) transporter inhibitor^6^; an antagonist for the 5‐HT_3_, 5‐HT_7,_ and 5‐HT_1D_ receptors; a partial agonist for the 5‐HT_1B_ receptor; and an agonist for the 5‐HT_1A_ receptor. Although no direct comparison standard exists, a lower incidence of jitteriness/anxiety syndrome is expected.^7^ In this report, we present three cases of patients who presented with jitteriness/anxiety syndrome after starting escitalopram but could be safely treated with vortioxetine, yielding improvement in depression.

## CASE PRESENTATION

### Patient A

The patient is a man in his 20s who has an aunt diagnosed with bipolar disorder. Two years after he graduated from college and started working, he began feeling fatigued and lost his appetite. He took some time off and recovered spontaneously. In his fifth year of employment, he began to experience depressive moods and thoughts of death. He visited a doctor and was diagnosed with major depressive disorder with a Beck Depression Inventory‐II (BDI‐II) score of 42 points. At this point, Patient A met the diagnostic criteria for major depressive disorder; however, because he was younger than 25 years of age at onset and had experienced more than two episodes per year, it was anticipated that he would transition to bipolar disorder^8^ in the future. Despite treatment with quetiapine and lithium carbonate, his symptoms did not improve. Three months after the start of treatment, the prescription of 10 mg escitalopram resulted in an improvement in activity and a decrease in his BDI‐II score to 26, although fluctuations in symptoms remained. Three months after the start of escitalopram, mood elevation, talkativeness, and increased activity emerged, and his daily life became unstable. Escitalopram and lithium carbonate were temporarily discontinued sequentially to reevaluate drug efficacy. Based on the patient's response, it was determined that lithium carbonate was ineffective, whereas escitalopram reduced depression but also induced intermittent mood elevation and increased activity. Two months later, escitalopram was replaced by vortioxetine, and the mood elevation ceased. Thereafter, his symptoms of depression gradually improved, and his BDI‐II score decreased to 12. The patient's mood swings did not recur within the next year, and he continues to work.

### Patient B

The patient is a woman in her 20s. She had been employed after graduating from high school, but she had developed emotional instability in her early 20s, which was triggered by relationship problems. She was diagnosed and treated for an adjustment disorder, and consulted with a doctor regarding her depressive mood and emotional instability. Insomnia and loss of interest were also noted, with a BDI‐II score of 33. Treatment was initiated with escitalopram. However, by day 6, she exhibited elevated mood, hyperactivity, irritability, and talkativeness. These symptoms disappeared within 24 h and her depression flared up. Escitalopram was discontinued, and she did not respond to treatment with aripiprazole, lurasidone, or lamotrigine. Three months after treatment initiation, she took a leave of absence from work due to the worsening of her depressive mood and suicidal ideation. She began treatment with vortioxetine, and her depressed mood had improved by the second week. By the third week after vortioxetine treatment, her BDI‐II score had lowered to 23 points, and further improved to 11 points by the sixth week. By the seventh week, she was able to return to work. Her excessive emotional instability disappeared, and her BDI‐II score was later reduced to 4. Since then, she has continued to work without any recurrence for at least 9 months.

### Patient C

The patient is a female in her late teens. She was admitted to a psychiatric hospital due to symptoms of depressed mood, anxiety, and death ideation during her junior high school years. She was diagnosed with bipolar disorder after being evaluated for an elevated mood and was subsequently treated with valproic acid, lithium carbonate, and olanzapine. While there was a period of symptom relief, her depression eventually relapsed and worsened. When treated with escitalopram, her depression, anxiety, and agitation were ameliorated. However, she exhibited irritability and self‐harm urges at the 2‐week evaluation of escitalopram treatment. Consequently, escitalopram was discontinued. Throughout this period, she frequently had difficulty attending school. Three months later, she was referred to our hospital. Upon admission, she exhibited anxiety and cried, and had a Hamilton Depression Rating Scale (HAM‐D) score of 14 points. Based on her presentation, her diagnosis was revised to depressive disorders with anxious distress, and she was treated with vortioxetine. By the second week, her HAM‐D score was reduced to 6 and her symptoms had stabilized. After discharge from the hospital, the patient continued to attend school.

**Figure 1:**
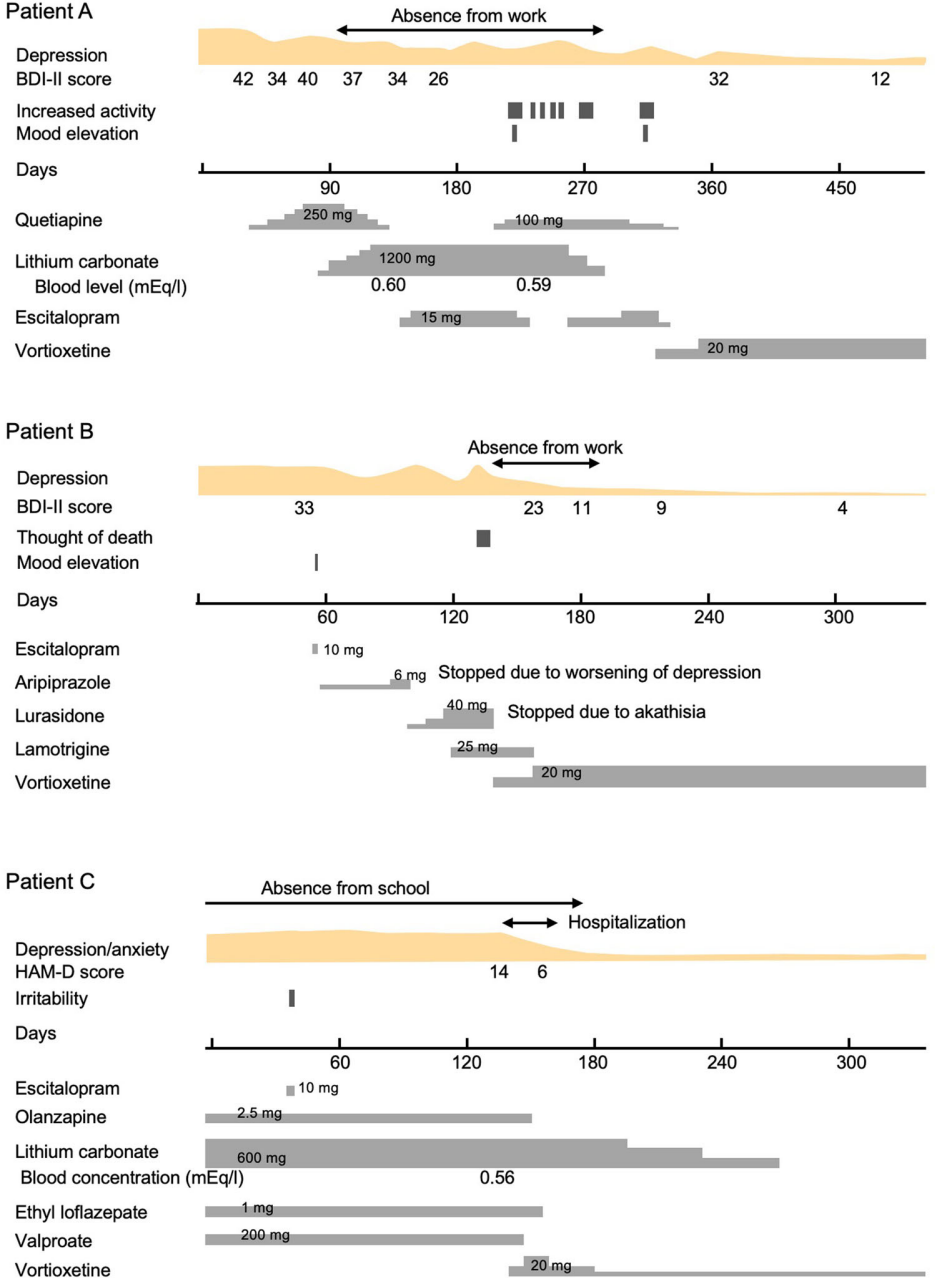
Time course of symptoms and treatment of each described case, and maximum dose of each administered drug. BDI‐II, Beck Depression Inventory‐II; HAM‐D, Hamilton Depression Rating Scale.

The clinical course of each case, including the details of pharmacotherapy, is shown in Figure 1.

## DISCUSSION

This report presents three patients who were treated with vortioxetine and did not experience the jitteriness/anxiety syndrome associated with escitalopram treatment. Although these patients required antidepressant treatment, they did not respond to mood stabilizers or antipsychotics and they had difficulty continuing escitalopram due to elevated mood, increased activity, and irritability. Although the benefits of vortioxetine have been noted and include improved cognitive function^9^ and low dropout rates,^10^ ours is the first report to offer new insight into the potential use of vortioxetine for the management of antidepressant‐induced jitteriness/anxiety syndrome that complicates treatment.

The jitteriness/anxiety syndrome experienced by the three patients differed in the symptoms presented and the timing of the presentation. Patient A experienced hypomania that appeared 3 months after the introduction of escitalopram. Patient B experienced transient mood elevation, hyperactivity, and irritability on day 6 of escitalopram medication, which lasted <24 h. Patient C experienced agitation and irritability, which occurred within 14 days of escitalopram treatment. These are discussed individually in this section.

Most symptoms of the jitteriness/anxiety syndrome appeared within 2 weeks of antidepressant initiation; however, only mania/hypomania was present in the second month.^11^ Therefore, some researchers have postulated that different mechanisms are responsible for mania/hypomania and jitteriness/anxiety.^4^ The hypomania exhibited by Patient A is consistent with previous reports of antidepressant‐induced hypomania/mania (AIHM). There are opinions that AIHM shows clinical features similar to bipolar disorder and should be categorized as such,^12^, ^13^ or that the correct diagnosis for depressed patients who experience AIHM is bipolar disorder.^14^ However, Patient A did not respond to treatment for bipolar disorder, and his symptoms decreased after the addition of vortioxetine to his treatment plan. It is suggested that AIHM is a heterogeneous condition; accordingly, only some patients respond to mood stabilizers. Vortioxetine may be useful for patients who present with AIHM but respond to antidepressants.

Patient B presented with short‐term elevated mood, increased activity, talkativeness, and irritability within 1 week of starting escitalopram. Because these symptoms were absent before escitalopram treatment was started, they can reasonably be considered antidepressant‐induced. Although the symptoms are similar to those of hypomania, the duration of their presentation is too short to meet the diagnostic criteria. In addition, the fact that the symptoms manifested immediately after the initiation of antidepressant medication differs from the typical timing of the appearance of AIMH as described above. Considering these characteristics, Patient B's condition is even more distant from bipolar disorder than Patient A's symptoms. Indeed, Patient B did not improve when she received treatment similar to that used for bipolar disorder.

Patient C presented with irritability. Symptoms such as irritability, aggression, and impulsivity have been reported to occur more frequently during antidepressant treatment in pediatric patients with a family history of bipolar disorder.^15^ No treatment strategy for this symptom has been established, and it has been suggested that the discontinuation of antidepressants may be advisable.^5^ Patient C's symptoms, which had appeared following the initiation of antidepressants, disappeared when escitalopram was discontinued. Patient C did not respond to mood stabilizers or antipsychotics, and vortioxetine eventually improved her symptoms.

All three patients that we have described here were in their 20s or younger. The relationship between the risk of developing jitteriness/anxiety syndrome and age is inconclusive; indeed, several studies of adult patients have shown that age was not a predictor of jitteriness/anxiety syndrome.^3^, ^16^ In the case of pediatric patients, there is evidence to suggest that it is more likely to occur in children aged 12 years and below, compared with those aged 13–17 years.^17^ In the case of the present study, we suggest that the small sample size involved precludes forming new insights into the epidemiology of antidepressant‐induced jitteriness/anxiety syndrome.

It has been estimated that vortioxetine has a 5‐HT receptor occupancy exceeding 80% at a 20‐mg oral dose,^6^ which is not significantly different from that of escitalopram.^18^ However, it is possible that the multimodal activity of vortioxetine suppresses jitteriness/anxiety. The notable pharmacological effects of vortioxetine are described as follows. Positron emission tomography (PET) studies in animals^19^ and humans^20^ show that 5‐HT_1A_ agonists inhibit aggression. Other studies evaluating the association between the serotonin receptor 1B (HTR1B) rs6296 genotype and childhood behavior^21^ suggest that the 5‐HT_1B_ receptor modulates aggression and hostility. Animal studies have shown that knocking out the 5‐HT_1B_ receptor increases aggressive behavior,^22^, ^23^ and that 5‐HT_3_ antagonists suppress aggression,^24^, ^25^ but contradictory results also exist depending on the pathogenesis of aggression.^26^, ^27^ Although a decrease in the 5‐HT_2_ receptor has been suggested in patients with mania,^28^ there remains a paucity of information on the involvement of the 5‐HT system in the context of mania and hypomania.

There are several limitations to the present report. The first is the heterogeneity of symptoms among the three cases reported here. As noted earlier, it is inconclusive whether mania/hypomania can be treated in the same way as jitteriness/anxiety. Second, although jitteriness/anxiety syndrome is a common adverse event in antidepressant treatment, the present study included only patients with jitteriness/anxiety syndrome caused by escitalopram. Further studies are required to clarify if switching to vortioxetine from antidepressants other than escitalopram is an effective treatment strategy for patients with antidepressant–induced jitteriness/anxiety.

It should be noted that this study does not advocate for the indiscriminate use of vortioxetine. Similar to other antidepressants, the vortioxetine package insert^29^, ^30^, ^31^ warns of the risk of inducing symptoms equivalent to antidepressant‐induced jitteriness/anxiety syndrome. Clinicians should follow this warning and monitor their patients carefully. In addition, we acknowledge the emerging literature pointing towards vortioxetine's potential risks of inducing hypomania, as reported in several case studies.^32^, ^33^, ^34^, ^35^, ^36^, ^37^ The efficacy of vortioxetine for treating pediatric patients with depression has not been confirmed. Therefore, its use is not approved in the United States or the European Union and is only permitted in Japan after careful consideration. The decision to administer vortioxetine in this study was made based on the authors’ experience and successful outcomes in similar prior cases. However, this does not negate the necessity for clinicians to exercise caution and thorough evaluation before prescribing vortioxetine.

## CONCLUSION

This report describes three patients who presented with jitteriness/anxiety syndrome during treatment with escitalopram for depression and could be safely treated with vortioxetine. Switching to vortioxetine may be worth considering for patients with jitteriness/anxiety syndrome who need antidepressants.

## AUTHOR CONTRIBUTIONS

Kaoru Arai drafted the manuscript. All authors were directly or indirectly involved in the patient's treatment and presented clinical history. Masayuki Nakamura reviewed and edited the manuscript. All authors read and approved the final manuscript.

## CONFLICT OF INTEREST STATEMENT

Masayuki Nakamura received an honorarium from Takeda Pharmaceutical Company Limited outside the submitted work.

## ETHICS APPROVAL STATEMENT

This study was conducted according to the Declaration of Helsinki.

## PATIENT CONSENT STATEMENT

Written consent was obtained from the patients.

## CLINICAL TRIAL REGISTRATION

This study is not registered because it is a retrospective record of medical practice.

## Data Availability

N/A.
